# Prevalence of hypospadias in Italy according to severity, gestational age and birthweight: an epidemiological study

**DOI:** 10.1186/1824-7288-35-18

**Published:** 2009-06-27

**Authors:** Paolo Ghirri, Rosa T Scaramuzzo, Silvano Bertelloni, Daniela Pardi, Amerigo Celandroni, Guido Cocchi, Roberto Danieli, Luisa De Santis, Maria C Di Stefano, Orietta Gerola, Mario Giuffrè, Giuseppe S Gragnani, Cinzia Magnani, Cristiano Meossi, Ilaria Merusi, Giuseppe Sabatino, Stefano Tumini, Giovanni Corsello, Antonio Boldrini

**Affiliations:** 1 Department of Paediatrics, Division of Neonatology and Neonatal Intensive Care Unit – "S. Chiara" Hospital, University of Pisa, Italy; 2 Department of Paediatrics, Section of Adolescent Medicine Unit – "S. Chiara" Hospital, University of Pisa, Italy; 3 Division of Pediatrics, "G. Pasquinucci" Paediatric Hospital, Massa; 4 Division of Pediatrics, – "F. Lotti" Hospital of Pontedera, Italy; 5 Division of Pediatrics – University of Bologna, Italy; 6 Division of Pediatrics, Hospital of Livorno, Italy; 7 Division of Pediatrics and Adolescent Medicine – "S. Anna" Hospital, University of Torino, Italy; 8 NICU – "Umberto I" Hospital, Nocera Inferiore, Italy; 9 Division of Pediatrics – IRCCS "S. Matteo" Hospital, University of Pavia, Italy; 10 Division of Pediatrics – University of Palermo, Italy; 11 Division of Pediatrics, Bassa Val di Cecina Hospital; 12 Division of Neonatology – University of Parma, Italy; 13 Division of Pediatrics, "Campo di Marte" Hospital, Lucca, Italy; 14 Division of Pediatrics, Versilia Hospital, Italy; 15 NICU and Division of Pediatrics – University "G. D'Annunzio", Chieti, Italy

## Abstract

**Background:**

Hypospadias is a congenital displacement of the urethral meatus in male newborns, being either an isolated defect at birth or a sign of sexual development disorders. The aim of this study was to assess the prevalence rate of hypospadias in different Districts of Italy, in order to make a comparison with other countries all over the world.

**Methods:**

We reviewed all the newborns file records (years 2001–2004) in 15 Italian Hospitals.

**Results:**

We found an overall hypospadias prevalence rate of 3.066 ± 0.99 per 1000 live births (82.48% mild hypospadias, 17.52% moderate-severe). In newborns Small for Gestational Age (birthweight < 10^th ^percentile) of any gestational age the prevalence rate of hypospadias was 6.25 per 1000 live births. Performing multivariate logistic regression analysis for different degrees of hypospadias according to severity, being born SGA remained the only risk factor for moderate-severe hypospadias (p = 0.00898) but not for mild forms (p > 0.1).

**Conclusion:**

In our sample the prevalence of hypospadias results as high as reported in previous European and American studies (3–4 per 1000 live births). Pathogenesis of isolated hypospadias is multifactorial (genetic, endocrine and environmental factors): however, the prevalence rate of hypospadias is higher in infants born small for gestational age than in newborns with normal birth weight.

## Background

Hypospadias is a congenital displacement of the urethral meatus in male newborns, often associated to an incomplete development of the foreskin (prepuce) and abnormal penile curvature (recurvatum or hypospadias sine hypospadias). According to the exact localization of the meatus, hypospadias is named glandular or coronal (mild), penile (moderate), scrotal and perineal (severe). While most cases of mild forms are usually an isolated defect, the severe ones may be a symptom of a disorder of sexual differentiation [1]. According to the Chicago Consensus Conference, severe isolated hypospadias can be classified either into among 46, XY Disorders of Sexual Differentiation ("Disorders in androgen synthesis or action") when it is supposed to be due to a complex disorders as 5α reductase deficiency or defects in androgen action, or classified into the C group ("Other"), when it is considered an isolated embryological defect [2].

The isolated malformation is likely caused by failed seam of the urethral folds at midline in the embryo. Patients need surgical repair, since they suffer from urological disorders and may have subfertility.

So far only few studies reported the prevalence rate of hypospadias in Italy, but they all were based on birth defect registries or referred to particular districts in Sicily, where pollution level is actually high [3-6].

We accurately assessed the prevalence rate and trends of hypospadias, including definition of different subtypes, in order to get exact data from several Italian districts for comparison with other countries all over the world.

## Methods

**Figure 1:**
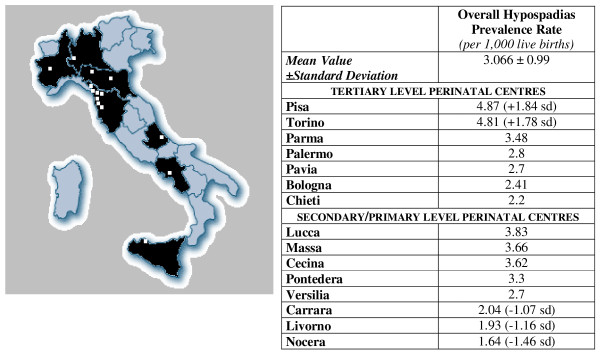
**The figure shows all the hospitals included in the study**.

We reviewed all the newborns file records, years 2001–2004, in 15 Italian Hospitals, namely: Bologna, Chieti, Palermo, Parma, Pavia, Pisa, Torino (tertiary level perinatal centers), Carrara, Cecina, Livorno, Lucca, Massa, Nocera Inferiore, Pontedera, Versilia (secondary or primary level perinatal centers). As shown in figure 1, we included Hospitals placed in different Districts of Italy, so that we could obtain a significant overall count.

The systematic inspection of all the neonates file records allowed us to avoid underreporting due to late or incomplete indications to local Registers of Congenital Malformations. By virtue of this method, we actually performed an accurate data collection. Unfortunately, we could not get the abortions file records: for this reason, we only calculated hypospadias rates among live births, instead of prevalence on all gestations.

Diagnosis of hypospadias was made at birth, by Paediatricians expert of neonatal dysmorphology. Since we collected data from hospital file records, that is defined as a Population type-2 Study by Eurocat, we had to check all cases of neonates twice admitted to NICU for different reasons. Moreover, we looked for cases of neonates moving from a hospital to another into those districts where Hospitals are organized according to the Hub & Spoke Model (that is primary, secondary and tertiary level perinatal centers).

In order to allow comparison with previously reported studies, we defined glandular and coronal hypospadias (first-degree) as mild, while penile hypospadias (second-degree) as moderate and scrotal and perineal hypospadias (third-degree) were reported as severe [7,8]. We focused on isolated hypospadias only, not considering cases of ambiguous genitalia or other compound genital malformations, classified as DSD other than 46, XY DSD-group C.

To the same purpose of comparison, hypospadias rates are finally presented as the number of cases per 1000 live births as well as in most studies already published.

Hypospadias rates are presented as mean ± standard deviation. Statistics was performed using the software R Project for Statistical Computing. For comparison of prevalence rates, we used χ^2 ^test for univariate analysis of categorical data, based on 2 × 2 cross-tabulations. We also performed Wilcoxon Rank Sum Test (Mann-Whitney) in order to assess risk factors separately. Finally, we used Multivariate Logistic Regression Analysis to show the likely relationship between hypospadias and pregnancy-related (i.e. maternal age, multiple gestation, parity, preterm birth, low birth weight) or environmental factors (i.e. hospital of birth).

The study was approved by the local Ethical Committee of all the Hospitals included.

## Results and discussion

Results are shown in table 1.

**Table 1:** Results explained in the text

	**HYPOSPADIAS PREVALENCE RATE (per 1000 live births)**	**P**
Overall rate	3.066 ± 0.99	

AGA (10^th^–90^th ^cent)	2.86	

SGA (< 10^th ^cent)	6.25	< 0.01


At term newborns (37–41 wks)	2.53	

Preterm newborns (< 37 wks)	6.34	


AGA at term	2.58	

SGA at term	5.28	< 0.01

We reviewed a total of 78228 consecutive newborns file records. Male/female ratio was 0.892.

**Figure 2:**
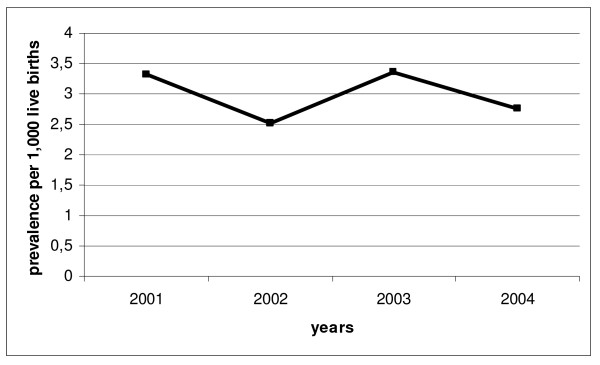
**The prevalence trend of hypospadias as we calculated in Italy in years 2001–2004**.

We assessed 234 cases of hypospadias among 36890 live boys, giving an overall prevalence rate of 6.34 per 1000 male newborns (2.99 per 1000 live births). As shown in figure 2, the prevalence trend was almost steady in the years 2001–2004.

Within this sample population of newborns with hypospadias, 82.48% had mild hypospadias while 17.52% moderate-severe, without significant differences of severity during the study period. However, an increasing trend in prevalence of moderate-severe forms was observed in tertiary level perinatal centres.

In newborns Small for Gestational Age (SGA, that is birth weight < 10^th ^percentile according to the normal tables for the Italian population [9]) of any gestational age the prevalence rate of hypospadias was 6.25 per 1000 live births, a figure considerably higher than in AGA (*Adequate for Gestational Age*) newborns (2.86 per 1000 live births) (p < 0.01).

In preterm newborns (gestational age < 37 weeks) the prevalence rate of hypospadias was 6.34 per 1000 live births vs 2.53 per 1000 live births in neonates of g.a. ≥ 37 weeks (p < 0.01). Within the sample of preterm newborns, we found a prevalence rate of hypospadias of 19.08 per 1000 live births in SGA neonates vs 3.83 per 1000 live births among AGA neonates (p < 0.001).

Finally, we calculated a prevalence rate of 5.28 per 1000 live births within SGA newborns born at term of pregnancy (g.a. ≥ 37 weeks), vs 2.58 per 1000 live births within the sample of AGA newborns at the same gestational age (p < 0.01).

According to these data based on univariate analysis, both preterm birth and small birthweight for gestational age seem to be risk factors for hypospadias. However, performing Wilcoxon Rank Sum Test (Mann-Whitney), we found that preterm birth and small birthweight for gestational age are associated one to each-other (p = 0.0134). For this reason, we performed logistic regression, in order to evaluate the possible perinatal risk associated with hypospadias. Multivariate logistic regression analysis was performed for risk factors, including SGA, preterm birth and hospital of birth. Only SGA remained significant in the final model for hypospadias (p = 0.00482).

Maternal age and multiple gestation were not related to hypospadias prevalence.

We also performed multivariate logistic regression analysis for different degrees of hypospadias, according to severity. In the final model SGA remained the only risk factor for moderate-severe hypospadias (p = 0.00898), but not for mild forms (p > 0.1).

## Conclusion

In our sample the general prevalence of hypospadias results as high as the value observed in previous European and American studies (3–4 per 1000 live births) (Table 2). The rates that we calculated are representative of different areas of Italy, since we collected data in 15 hospitals to a large extent evened out within the country.

**Table 2:** Comparison between our data and previous studies about hypospadias

	**YEARS**	**POPULATION** **(Reviewed file records/Selected patients)**	**CASES OF HYPOSPADIAS**	**PREVALENCE**
Our data	2001–2004 (Italy)	78228	234	3.066 ± 0.99 (per 1000 live births)

Pierik et al, 2002 *(Hum Reprod)*	1998–2000 (Olanda)	7292	53	3.8 (per 1000 live births)

Martti Olavi Aho et al, 2003 *(Envir Res)*	1970–1986 (Finlandia)	2164720	6,144	2.03–3.28 (per 1000 boys)

Bianca et al, 2003 *(Reprod Toxicol)*	1991–1998 (Sicilia, Italia)	1323 (Augusta) 3243 (Vittoria)	16 (Augusta) 24 (Vittoria)	12.1 (Augusta) 7.4 (Vittoria) (per 1000 boys)

Ahmed et al, 2004 *(Arch Dis Child-Fetal Neon Ed)*	1988–1997 (Scozia)	611849	2816 **	4.6 ** (per 1000 live births)

Porter et al, 2005 *(Pediatrics)*	1987–2002 (Washington State, USA)		2155	4.6 (year 1987) 5.0 (year 2002) (per 1000 live births)

* Data referred to SGA (Small for Gestational Age) newborns in NICU

** Data referred to genital abnormalities (hypospadias = 73%)

*** Data referred to hypospadias and epispadias

In the 1980s European registers reported upward trends in hypospadias prevalence: the rate almost doubled in Wales, Sweden, Norway and Hungary [8]. These changes could be due either to an actual increasing frequency of hypospadias occurence, or to the increasing identification of mild cases by physicians. In fact, looking at Eurocat reports, we noted that the main difference in prevalence rate (Table 3) is likely due to the exclusion of glandular hypospadias in most Registries connected to Eurocat [10]. However, the ratio of mild to severe cases did not increase as well as overall hypospadias prevalence rate [8] and this observation makes data more difficult to interpret. In fact, we can speculate that either prevalence of hypospadias actually increased (both mild and severe forms) or mild cases have been more frequently noticed while only severe forms percentage really increased, as well as reported by Nassar et al. in Western Australia [11].

**Table 3:** Comparison between our data and EUROCAT *(from Dolk et al. 2004)*

	**YEARS**	**POPULATION** **(Reviewed file records/Selected patients)**	**CASES OF HYPOSPADIAS**	**PREVALENCE (per 1000 live births)**
**Our data**	2001–2004	78228	234	3.066 ± 0.99

**Northern-Eastern Italy**	1981–1999	894344	554*	0.6

**Tuscany**	1980–1999	306517	233**	0.8

**Emilia-Romagna**	1981–1999	445289	281	0.6

**Sicily**	1991–1998	152237	237***	1.6

*Exclusion of glanular and coronal hypospadias

**Implementation of exclusion guideline only since 1992

***Not implementing guideline to exclude glanular hypospadias

We reported a steady trend of hypospadias in Italy in years 2001–2004 without any increase of severe forms.

Pathogenesis of isolated hypospadias is multifactorial, since genetic, endocrine and environmental factors have been implicated. These factors act in the early gestation disrupting the normal genitalia development in the male fetus, likely modifying androgen production or action [6]. Because of the recent changes in the environment and in pregnancy management, more studies about risk factors for urological anomalies and subfertility are needed, with special attention to those risk factor that may have become more prevalent during the last years (*in vitro *fertilization, pollution, medical techniques in neonates assistance, etc.). Effective recognition of endocrine disrupting properties of chemicals (acting estrogens or antiandrogens-like, i.e. phthalate esters) that developing fetuses and children are exposed to is the first step to prevent environmental causes of disorders of sexual differentiation, including isolated disorders as hypospadias [12].

We included in the study tertiary, secondary and primary level perinatal centers, so that we got data from both nurseries for healthy newborns and NICU for neonates with any problems. The prevalence rate of hypospadias seems to be higher in infants born small for gestational age (SGA) than in newborns with normal birth weight [13]. Our study confirms a higher prevalence of hypospadias among SGA newborns than AGA ones (Table 4). Preterm newborns have a higher prevalence too, but this result was confirmed in preterm SGA infants only, not confirmed in preterm AGA. This implies that either factors underlying the cause of hypospadias are also more likely to result in fetal growth retardation or the growth retarded fetus is more susceptible to influences causing hypospadias [14], while this conclusion is not consistent with factors causing preterm birth. However, preterm newborns are more frequently SGA than those born at term, so that data could be mixed up at a first analysis. Nevertheless, the actual cause of the observation linking fetal growth retardation to hypospadias remains undiscovered.

**Table 4:** Small for gestational age newborns with hypospadias

	**Our data**	**Gatti et al. 2001**
**Gestational age (median value)**	33 weeks	33.5 weeks

**Birthweight (median value) **[< 10^th^*cent*]	1340 g	1320 g

**Prevalence of hypospadias**	8.2%	11%

We found differences among Italian hospitals that may be mainly related to the percentage of SGA and preterm newborns. For example, among tertiary level perinatal centres, we assessed a hypospadias prevalence rate of 4.87 per 1000 live births in Pisa, while 2.2 per 1000 live births in Chieti, considering a number of SGA of 9.5 per 1000 live births in Pisa and 3.3/1000 in Chieti. Similarly, among secondary and primary level perinatal centres, we showed a hypospadias prevalence rate of 3.83 per 1000 in Lucca (SGA: 43 per 1000) and 1.64 per 1000 in Nocera (SGA: 5.6 per 1000).

Performing multivariate logistic regression analysis, we showed that SGA is a risk factor for all the different subtypes of hypospadias, even if the association is statistically significant only for severe ones. Because of this observation, we can speculate that mild and moderate hypospadias are likely due to environmental factors that disturb the pregnancy but not alter the whole growth of fetus. On the contrary, severe ones are probably correlate to genetics or important factors that are able to impair the general development of fetus-placental unit. In fact, few recently reported studies describe SGA newborns as a group at a major risk of severe birth defects.

However, we can also speculate about pollution and about the effects of exogenous hormones influencing development. Recent studies, in fact, report that maternal intake of progestins during early pregnancy may be associated with an increased risk of hypospadias [15]. Progesterone and its derivatives are commonly prescribed during early pregnancy, for example, in cases of luteal phase dysfunction and in conjunction with ovulation stimulation drugs. The different hypospadias prevalence rate that we found among Italian hospitals may be also related to different strategies in treating such gynaecological problems. Moreover, further studies could be interesting about incidence of conventional in vitro fertilization (IVF) and ICSI (intracytoplasmatic sperm injection) in these hospitals, since hypospadias has been reported to occur more frequently in children born after ICSI, maybe related to a paternal subfertility with a genetic background [16-20].

## Competing interests

The authors declare that they have no competing interests.

## Authors' contributions

All the authors participated in the data collection. GP and SRT designed the study, carried out the data elaboration and coordinated the study.
